# Atropine as an Antidote to Opium

**Published:** 1873-09

**Authors:** James Johnson

**Affiliations:** Medical Officer to the Chinese Hospital, Shanghai


### Atropine as an Antidote to Opium.

By Dr. James Johnson, Medical Officer to the Chinese Hospital, Shanghai.

Without entering into detailed remarks on individual cases, I may observe that, on every occasion when atropine was injected, the patient was at that time, or very shortly afterwards, in a state of the most profound coma, and utterly insensible to the cold douche, galvanism, or any other remedial measures, and the general condition was as follows:—Pupils firmly contracted to a pin's point, and immovable; conjunctiva and surface of cornea insensible to touch; face pale; lips, eyelids, and nails livid; pulse weak and irregular; respiration slow and stertorous; extremeties cold, and the state of the body generally such as to give it the appearance of a lifeless mass. It was in this condition that the wonderful power of atropine showed itself. The quantity ordinarily injected was half a grain, and its effects were, that in from ten to twenty minutes the pupils began to dilate slowly, and after an hour or more the face became flushed, the respiration soft without stertor, the pulse stronger. Within two hours the full effects of the drug were usually manifested—viz, widely dilated pupils, flushed face, hot skin, tranquil, slow breathing, diminished frequency and increased strength of pulse, followed by calm and tranquil sleep, from which the patient was easily waked after three or four hours. The indication for repeating the injection of atropine was the failure of the first dose to produce within two hours the dilated pupil, flushed face, and slow, steady, tranquil breathing. The dose injected varied according to the degree of coma; when very profound, half a grain was the usual dose; when only partial, a quarter of a grain, repeated afterwards if necessary. The action of the atropine was very uniform—dilating the pupil, strengthening and steadying respiration, and lessening the frequency and increasing the strength of the pulse. Of the fatal cases in which atropine was used, the first one reported lived for eighteen hours, and the action of the atropine was very manifest, as the patient partially recovered consciousness, and did not perish from the comatose effects of opium, but from exhaustion following that condition. In the second fatal case the patient was only two hours and a half under treatment, and died from cerebral apoplexy. The third was under treatment a little over two hours, and died from cerebral effusion. The fourth was two hours under treatment, and died from exhaustion. The fifth was under treatment for five hours, and died from convulsions. The sixth also died from convulsions, and was under treatment for three hours and a half. During the past seven years, upwards of three hundred cases of opium-poisoning have come under my observation in this Hospital. In the majority, where the patient was brought within an hour or two after swollowing the poison, the ordinary treatment—by emetics, stomach-pump (when required), cold douche, moving the patient about, stimulants and coffee—was successful; but in the worst of cases, when the hapless patient was in profound coma, and insensible to cold douche, galvanism, and artificial respiration, remedies were often of no avail, and the wretched suicide died.

In July, 1869, I first injected atropine subcutaneously in a very bad case of opium-poisoning, and although it failed to rescue the patient, I was so struck by its effects that I resolved to make a further trial of it. The class of cases in which the wonderful powers of atropine as an antidote to opium-poisoning are most marked, is where profound coma exists; and for such a condition I know no other remedy. You may try to drag the patient about, use the cold douche, carry on artificial respiration assiduously, give stimulants of every description; all in vain—the patient remains comatose and lifeless. Administer atropine, and the effects are marvellous: the pupils dilate; the face becomes flushed; the respiration loses its stertorous character and becomes slow and tranquil; the pulse is diminished in frequency and becomes stronger; the patient slumbers peacefully for several hours, and at last wakes up conscious. I have said that all the cases reported in which atropine was used were of a severe kind, and that the treatment by emetics, &c., in milder cases was usually successful; but in some instances I have observed very unfavorable changes set in very rapidly, even in the mildest cases of opium-poisoning. This has happened so frequently that I have come to the conclusion that whenever there is contraction of the pupil and great drowsiness after the evacuation of the contents of the stomach and the moving about of the patient for a little, it is always advisable to exhibit a small dose of atropine, so as to bring the system under its influence. I may remark that in no instance have I seen any bad effects following the subcutaneous injection of atropine. After recovery the patients complained of great lassitude, dryness of throat, double vision, &c., but they usually recovered from these symptoms very soon. The conclusions I have come to with reference to the treatment of cases of opium-poisoning are—

1. That in mild cases, when the patient is seen within one or two hours after taking the poison, and is conscious and able to move about, with pupils uncontracted and movable, the ordinary treatment by emetics, &c., will usually suffice for recovery; but the greatest watchfulness and care are necessary, for even in the mildest cases dangerous symptoms speedily set in. As a rule, whenever there is great drowsiness, with contracted pupils, after the evacuation of the contents of the stomach and moving about of the patient, it is advisable to bring the system under the influence of atropine at once.

2. The state of the pupils is of the first importance. Whenever they are firmly contracted to a pin's point, there is danger; although the patient for the time may seem but slightly affected, sooner or later coma inevitably comes on. It is, therefore, advisable in such cases to use atropine at once.

3. That in cases when the nervous centres do not respond to the cold douche and moving about the patient, it is not only useless, but mischievous, to persist in attempts to drag the individual about, as it only increases the exhaustion, which is one of the greatest dangers in cases of opium-poisoning. The treatment for such cases is to place the patient in the horizontal position, and inject the atropine subcutaneously, and, if necessary, assist with artificial respiration, and, in case of exhaustion, to strengthen the circulation by applying warmth and counter-irritation to the limbs, and by the administration of stimulants, such as coffee, ammonia, brandy, &c., internally.

4. That in all cases of profound coma from opium-poisoning, with perfect insensibility, firmly contracted pupils, and stertorous breathing, atropine should be exhibited at once, and the patient carefully supported afterwards by the administration of coffee, ammonia, and stimulants.

5. That, when the system is fairly under the influence of atropine, with respiration tranquil, however slow it may be, it is undesirable to interfere by artificial respiration, as it only embarrasses the breathing and interferes with the tranquil sleep which usually follows the exhibiton of atropine.—*Medical Times and Gazette.—Braithwaite's Retrospect.*

